# Sharing knowledge to advance healthcare policies in Europe for people living with dementia and their carers: the ALCOVE project

**DOI:** 10.1186/0778-7367-70-21

**Published:** 2012-08-28

**Authors:** Christine Barr, Nathalie Riolacci-Dhoyen, Maggie Galbraith, Armelle Leperre-Desplanques

**Affiliations:** 1 Haute Autorité de Santé (HAS), Avenue du Stade de France, 93218, Saint-Denis La Plaine, France; 2 ALzheimer Cooperative Valuation in Europe Group, c/o Executive Agency for Health and Consumers, Health Unit, DRB A3/050, L-2920, Luxembourg, United Kingdom

**Keywords:** Alzheimer’s disease and related dementia, Dementia, Public health, BPSD, Antipsychotics, Risk reduction, Prevalence, Diagnosis, Advanced declaration of will, Health care system

## Abstract

****Background**:**

Alzheimer’s disease and other related dementias are public health priorities in the European Union due to their prevalence, cost and profound impact on society. Because of these pressing implications, the European Union decided to create a Joint Action to share knowledge about dementia and health policy in order to preserve the health, quality of life, autonomy and dignity of people living with dementia and their carers in Europe.

****Methods**:**

ALCOVE is a European Community-funded Joint Action coordinated by the HAS (French National Authority for Health) with a 24-month duration. The project’s life cycle has been divided into the following four steps: (1) collection of existing information, (2) analysis of existing information and making comparisons across Member States, (3) identifying Evidence, Needs, and Priorities, (4) drafting recommendations and disseminating them.

****Results**:**

19 countries are participating in the ALCOVE initiative. The project will publish its final findings in 2013. The project’s objectives, participants, method, on-going procedures and work plans are already available on the ALCOVE website: http://www.alcove-project.eu/. Preliminary results show that recommendations will need to focus on clinical and epidemiological data collection, diagnostic system assessment, outstanding approaches for treating behavioural disorders, limiting antipsychotic use, and competence assessment in this vulnerable population.

****Conclusions**:**

The European Member States involved are mobilized to share best health policy practices in order to tackle the challenge of dementia’s threat on European health and social systems and to improve the quality of life and care for individuals and their family carers.

## Background

The last 50 years in Europe have seen an overall dramatic increase in life expectancy, with a corresponding rise in the incidence of diseases linked to aging such as dementia [1,2]. More than 7 million Europeans are directly affected by dementia, the most common form being Alzheimer’s disease which is characterised by progressive memory loss and cognitive troubles as well as the occurrence of disturbing behaviour disorders [3,4]. Alzheimer’s disease and related dementias are public health priorities in EU Member States due to their prevalence, cost and profound impact on society [5-9].

**Figure 1:**
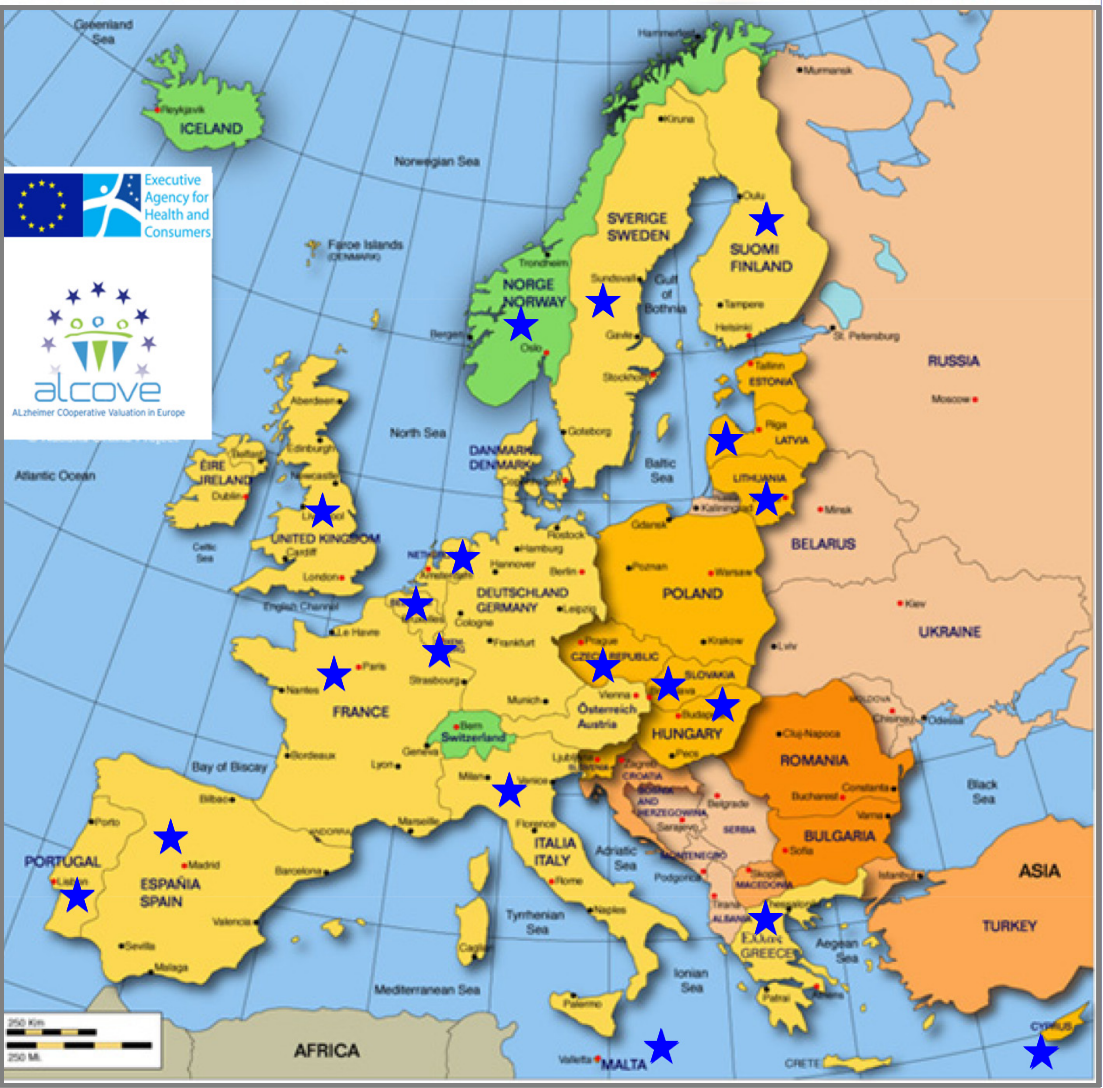
**European Union Joint Action ALCOVE.** (Alzheimer COoperative Valuation in Europe), 19 participating countries.

ALCOVE is a European Community-funded Joint Action (JA) coordinated by the HAS (French National Authority for Health), with the participation of 19 European Member States (MS) and involving 30 partner organisations (Figure 1, Additional file 1: Annex 1). The organisations have been designated by their national governments for their clinical, epidemiological or public health expertise in Alzheimer’s disease and related dementias.

The project aims to share knowledge about dementia and to preserve the health, quality of life, and the autonomy and dignity of people living with the disease and their carers in European Union Member States. To this end, the ALCOVE project aims to establish a sustainable European network (1) to provide better care and services all along the progression of Alzheimer’s disease and related dementia from early diagnosis to home or institutionalized care, including reduction in the risk related to the use of antipsychotics [10-13]; (2) to exchange experiences and knowledge about dementia health policy [13-15]; and (3) to inform and advise decision-makers, healthcare professionals, carers, and citizens.

The goal of the ALCOVE-Joint Action is to reply to the following strategic questions in order to guide public health decisions in EU Member States by fostering better coordination of the management of care for people living with Alzheimer’s Disease (AD) and other related dementias:

1. How to improve data for better knowledge about dementia prevalence?

2. How to improve access to a dementia diagnosis and, in the process, to the appropriate care as early as possible?

3. How to improve care for people living with dementia and particularly those exhibiting the Behavioural and Psychological Symptoms of Dementia (BPSD)?

4. How to improve the rights of people with dementia regarding advance declarations of will and competence assessments?

The use of antipsychotics in this vulnerable population has been chosen as a common ground for the 4 core questions and as a guide to support the implementation of good practices in the field of risk prevention associated with the use of psychotropic drugs in people living with dementia [12,13,16].

## Methods

**Figure 2:**
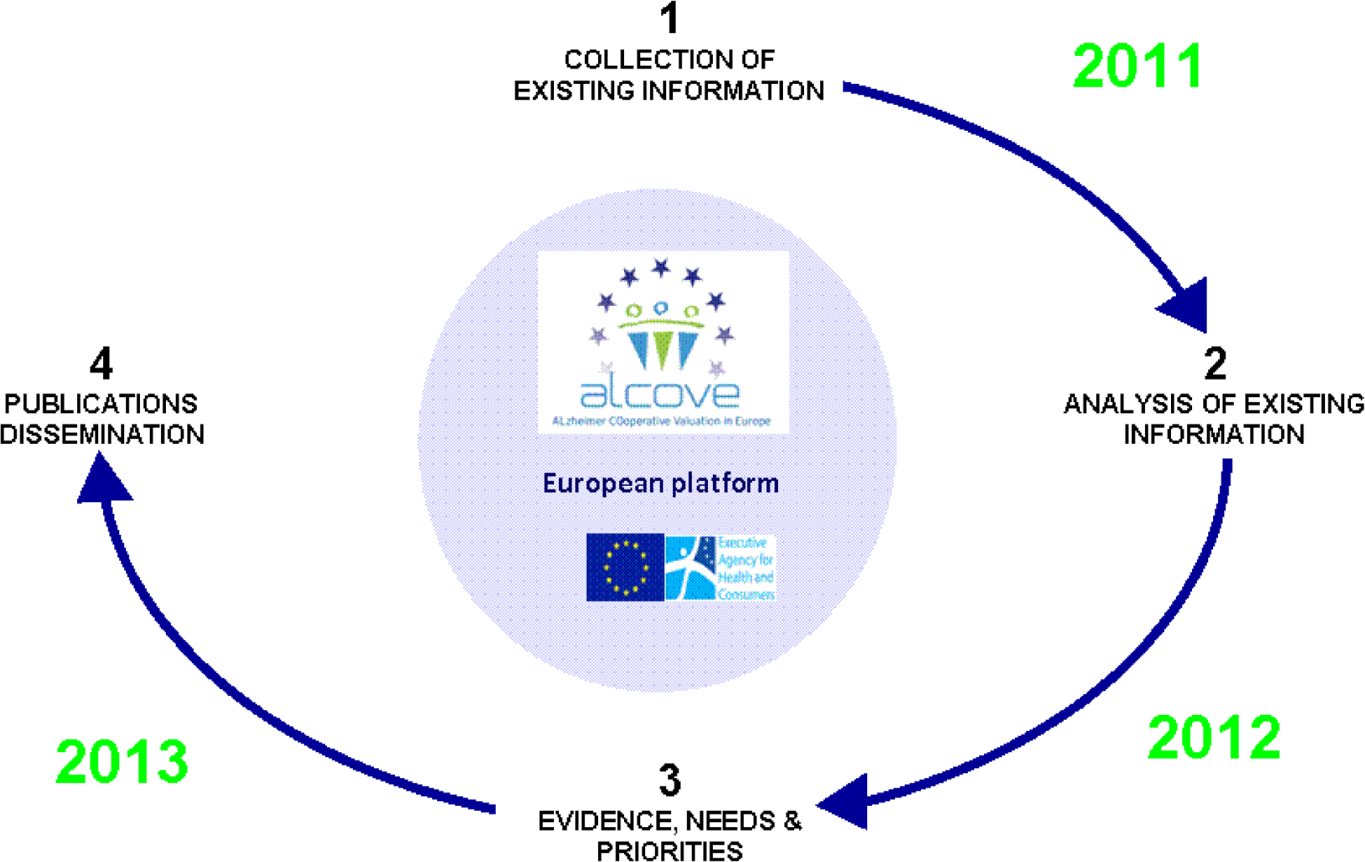
ALCOVE Collaborative Method.

ALCOVE is an operational public health project with a 24-month duration. The project’s life cycle has been divided into four steps as described below. ALCOVE is currently working on steps 1 and 2 and has already begun collecting the existing information on available epidemiological data in Europe and on the most recent scientific findings regarding good practices in the care of individuals living with Alzheimer’s disease and related dementia. The project’s next steps will be to identify and prioritize the needs of people living with Alzheimer’s disease and related dementia and to issue recommendations on how to best address these needs. The process that ALCOVE is following is highlighted in Figure 2[17].

1. Collection of existing information

ALCOVE’s method entails reviewing recent scientific literature, with an explicit strategy: period and terms of research, sources of information [Automated bibliographic databases; Medline (National Library of Medicine, United States), the Cochrane Library (Wiley Interscience, United States), OVID, In-Process & Other Non-Indexed Citations and Ovid MEDLINE; Pascal; CLEIRPPA database (FNG), French National Geriatrics; National Guideline Clearinghouse (United States); HTA Database (International Network of Agencies for Health Technology Assessment - INAHTA), Guidelines International Network]. Additionally, a grey literature search was conducted to identify Alzheimer Plans that have been implemented at the national level and evaluations of such plans. To this end, the project has consulted and used relevant websites, such as those of learned institutions specializing in the studied field, as well as the bibliographies of the selected articles and documents.

This literature review is being combined with the use of ALCOVE-designed questionnaires (Additional file 2: Annex 2) to collect relevant information from principal actors in the field such as National Health Ministries, Physicians, Geriatricians, and Memory Clinics as well as National Health Registries and databases.

2. Analysis of existing information and making comparisons across Member States

ALCOVE’s first aim is to improve data for better knowledge about dementia: the research is focused on prevalence rates for Alzheimer’s disease and related dementia in Europe and the evaluation of the rate of exposure of Europeans living with dementia to antipsychotics.

In an effort to understand differences across MS in the timely access to a dementia diagnosis, ALCOVE will compare national recommendations for a timely diagnosis in order to establish a common definition. ALCOVE will investigate the organisation of diagnostic systems within Member States and the localisation and availability of Memory Clinics while examining the role of General Practitioners and strategies for a timely diagnosis in Primary Care, including nursing/retirement homes.

In order to improve care for people living with dementia, and particularly those experiencing behavioural disorders, ALCOVE will identify the existing support systems for those exhibiting BPSD, specifically focusing on innovative and/or efficient measures in limiting the prescription of antipsychotics to treat BPSD, which have recently been shown to be non- effective and deleterious for these patients [12,13].

ALCOVE will analyze the current education that health care personnel who work with patients exhibiting BPSD receive and highlight areas where this education could be enriched. Furthermore, ALCOVE will be collecting data on the procedures currently in place for the prescription of antipsychotics (the majority have an off- label use) in clinical practice within European countries.

Within the context of ALCOVE’s fourth question regarding improving the autonomy of individuals living with Alzheimer’s disease and related dementia, the project will be identifying the existing national regulations on advance directives of will and informed consent, investigating the possibility of moving towards a more coordinated European perspective, describing how advance directives and informed consent are used in practice for people living with Alzheimer’s disease and related dementia, and identifying the good practices for competence assessment while documenting how the prescription of antipsychotic drugs could affect their validity.

3. Evidence, needs, and priorities

Based on the analyses and the answers to the four main questions, attained through the combination of literature searches, and the analyses of evidence and assessed practices or systems, ALCOVE will be able to both identify (a) the systems or practices devoted to dementia in place in the different European Union Member States, including how much they differ or vary from one another; and (b) the good practices and support systems which could be promoted at the European level.

4. Recommendations and Dissemination

Finally, ALCOVE will be making recommendations concerning good practices in epidemiological data collection, in the organisation of health systems or services for an earlier diagnosis, in the support systems and educational programmes for preventing BPSD and the overuse of antipsychotics, in competence assessment and the drafting of advanced declarations of will for this population.

Dissemination of our findings and analyses will be assured by a dedicated website, public and scientific conferences, professional publications and cooperation with other international referent networks.

## Results and discussion

The ALCOVE JA is an on-going project that will not publish its findings until 2013. As such, it is not generating original research, but rather is analyzing recent scientific findings and current treatment and care realities in Europe so that recommendations can be generated for good practices in diagnosing and treating individuals living with Alzheimer’s disease and related dementia, both at a theoretical and scientific level. The project’s objectives, participants, method and on-going procedures and workplans are already available on the ALCOVE website: ( http://www.alcove-project.eu/).

ALCOVE preliminary results show:

(1) That currently there is a discrepancy between the observed rate of prevalence in the national health systems, when this data is available, and the observed rate in cohorts. An updated mapping of different sources such as national insurance databases, registries, cohorts, surveys and recommendations to improve the quality and coherence of data collection could contribute to a better knowledge of the prevalence of Alzheimer’s disease and related dementia, which is crucial in the construction of relevant strategies. [5,6]

(2) That Alzheimer’s disease and related dementia are under-diagnosed all across European Union Member States. At this stage, improving diagnosis quality means making a timely diagnosis of the disease. Several European countries have already been working with memory centres for 10 years and the first appraisals of their effectiveness are now available. [18,19]

(3) That delivering better care in the treatment of BPSD is a shared and high level priority. BPSD is a large burden for daily carers and health professionals, so much so that it is a major reason behind early institutionalisation and/or the prescribing of antipsychotics. Currently, new non-pharmaceutical interventions are being implemented in an effort to reduce BPSD and their consequences, such as targeted care techniques and psychological and environmental interventions. The assessment of these non-pharmaceutical approaches is still on-going. [14,20-24]

(4) Various and heterogeneous legal dispositions for people living with dementia are available in European Union Member States. An important question is how to find a better balance between preserving autonomy while legally protecting the individual (driving licence, will). Health professionals need dedicated information and training on good practices for competence assessment in people living with dementia. [25-31]

(5) ALCOVE’s participants have decided that a special focus will be made on the inappropriate use of antipsychotics to treat the BPSD of Alzheimer’s disease and related dementia as this is an urgent issue affecting patient safety and the quality of life of individuals living with dementia and their carers [12-14,16] As the culmination of the project, ALCOVE will provide information on the exposure of patients living with dementia to antipsychotics in Europe and an evaluation of the existing antipsychotics risk reduction strategies for people living with dementia.

An operational set of improvement tools and methods (information support, indicators, professional programmes, clinical guidelines, etc) based on the European benchmarking performed by ALCOVE will be proposed and will be available on ALCOVE website ( http://www.alcove-project.eu/).

## Conclusion

ALCOVE’s partners and work package leaders share the concrete goal of ensuring patient safety and quality of life for individuals living with Alzheimer’s disease and other dementias in Europe. An important element of this goal and the tie that binds the work being completed by all of those involved is a focus on reducing the overexposure to antipsychotic drugs among individuals living with dementia. In compiling the most recent research and evidence of good practices already implemented in the care of dementia, it is ALCOVE’s mission to share this information so that Europeans living with Alzheimer’s disease will have access to new care options from which they will benefit.

By promoting the best options in Alzheimer’s disease care and providing a toolbox of care options, ALCOVE will help European Member States reduce the risks involved in the use of antipsychotic medications to treat BPSD, promote alternative treatments, and alert and educate carers so that they will be better equipped and more supported when caring for individuals living with Alzheimer’s disease.

## Endnote

ALzheimer COoperative Valuation in Europe.

## Competing interests

The authors declare that they have no competing interests.

## Authors’ contributions

CB, NRD, MG, and ALD were all involved in drafting the manuscript and the AG was involved in drafting the questionnaires, collecting, and analysing the data. All authors were involved in the critical revision of the manuscript. All authors read and approved the final manuscript.

## Authors’ information

***ALCOVE GROUP****:* Dr Armelle Leperre – Desplanques, Dr Nathalie Riolacci – Dhoyen, Christine Barr, Maggie Galbraith, Haute Autorité de Santé, France; Tomás López-Peña Ordoñez, Carlos Segovia, Gloria Villar Acevedo, Institudo de Salud Carlos III, Spain; Pr Michal Novak, Pr Rostislav Skabranova, Martina Jerzovicova, Slovenska Akademia Vied – Neuroimmunologicky Ustav, Slovakia; Pr Nicola Vanacore, Pr Francesca Galeotti, Pr Angela Giusti, Pr Fiorentino Capozzoli, Istituto Superiore di Sanita, Italy; Dr Karim Saad, Regional Clinical Lead for Dementia, Coventry UK; Pr Dawn Brooker; Dr Simon Evans, Dr Jerry La Fontaine, University of Worceter, UK; Jerry Bird Department of Health, UK; Dr Helka Hosia-Randel, Pr Matti Mäkelä, Harriet Finne-Soveri, Paivi Topo, Ulla Eloniemi-Sulkava, National Institute of Health and Welfare, Finland; Bénédicte Gombault, Gerrit Raws, Tom Goffin, King Baudoin Fundation, Belgium; Dr Catherine Helmer, INSERM, France; Pr Anders Wimo, Karolinska Institute, Sweden; Areti Efthymiou, Eleni Margioti, Nikolaou Costas, Maria Panagiotou, Paraskevi Sakka, Athens Association of AD and Related Disorders, Greece; Teresa di Fiandra, Giovanni Nicoletti, Fabrizio Oleari, Fiammetta Landoni, Cecilia Prezioso, Ministero della Salute, Italy; Barbara Borroni, Luca Rozzini, Enza Castronovo, Alessandro Padovani, University of Brescia, Neurology Clinic, Italy; Pr Wladimir Kuznecovs, Riga Centre of Psychiatry and Addiction Disorders, Latvia; Pr Daiva Rastenyte, Kaunas University of Medicine, Lithuania; Pr Gemma Villanueva, Pr Gemma Lopez Argumendo, Fundacion Vasca de innovation e investigation Sanitarias, Spain; Dr Gregory Emery, Virginie Ponelle, Pr Emmanuel Hirsh, Espace Ethique de Assistance Publique des Hôpitaux de Paris, France; Irene K-Georghiou, Health Services, Ministry of Health, Cyprus; Martina Mátlová, Ceska alzheimerovska spolecnost, Czech Republic; Janos Kalman, University of Szeged - Szegedi Tudományegyetem, Hungary, Genovaite Paulauskiene, Ministry of Health, Lithuania; Valmantas Budrys, Vilnius University Faculty of Medicine, Lithuania; Dorothee Knauf-Hübel, Ministère de la Santé, Luxembourg; Malou Kapgen, Ministère de la Famille et de l'Intégration, Luxembourg; Isabelle Avallone, Ministeru tas-Saħħa, l-Anzjani u l-Kura fil-Kommunita, Malta; Jacqueline Hoogendam, Ministry of Health, Welfare and Sports, Netherlands; Kristin Løkke, Ministry of Health and Care services, Norway; Miguel Xavier, National Coordinating Body for Mental Health, Portugal; Lubica Pitlova, Ministerstvo školstva Slovenskej Republiky, Bratislava, Slovakia; Isabel Saiz, Spanish Ministry of Health, Spain; Louise McCabe, Dementia Services Development Centre, University of Sterling, UK.

## Supplementary Material

Additional file 1 **Annex 1.** European Union Joint Action ALCOVE (Alzheimer COoperative Valuation in Europe) partners. Click here for file

Additional file 2 **Annex 2.** ALCOVE QUESTIONNAIRES. Click here for file
