# Supplementary material for: Sharing knowledge to advance healthcare policies in Europe for people living with dementia and their carers: the ALCOVE project
Source: Arch Public Health. 2012 Aug 28;70(1):21. doi: 10.1186/0778-7367-70-21 (PMC3523028; doi:10.1186/0778-7367-70-21)
Supplement: Additional file 1 — Annex 1. European Union Joint Action ALCOVE (Alzheimer COoperative Valuation in Europe) partners. [file 0778-7367-70-21-S1.doc]

Annex 1: European Union Joint Action ALCOVE (Alzheimer COoperative Valuation in Europe) partners

Belgium [King Baudouin Foundation, WP7 Leader]; Cyprus [Mental Health Services, Ministry of Health*]; Czech Republic [Ceska Alzheimerovska Spolecnost*]; France [Haute Autorité de Santé, WP1 leader, Coordinator; Assistance Publique-Hôpitaux de Paris, Espace Ethique; Institut National de la Santé et de la Recherche Médicale]; Finland [**Institute of Health** **and Welfare, WP6 Leader**]; Greece [Athens Association of Alzheimer’s Disease and Related Disorders]; Hungary [Szegedi Tudomanyegyetem*]; Italy [**Istituto Superiore di Sanità, WP4 leader**, Ministry of Health, Prevention Directorate; Università degli Studi di Brescia]; Latvia [Riga Center of Psychiatry and Addiction Disorders]; Lithuania [Vilniaus Universiteto Medicinos Fakulteto*; Lietuvos Respublikos Sveikatos Apsaugos Ministerija*; Lithuanian University of Health Sciences]; Luxemburg [Ministère de la Famille et de l’Intégration*; Ministère de la Santé*]; Malta [Ministeru tas-Sahha, l-Anzjani u l-Kura fil-Kommunitta*]; Netherlands [Ministry of Health, Welfare and Sport*]; Norway [Ministry of Health and Care Services*]; Portugal [Coordenação Nacional de Saude Mental, Alto Comissariado para a Saude, Ministerio da Saude*]; Slovakia [**Institute of** **Neuroimmunology**, **WP3 leader**, Slovak Academy of Sciences; Ministerstvo Skolstva Slovenskej Republiky*]; Spain [Instituto **de Salud** **Carlos III, WP2 Leader**; Spanish Ministry of Health*; Fundacion Vasca de Innovacion e Investigacion Sanitarias]; Sweden [Karolinska Institutet]; UK [**Department of Health, WP5 Leader**; University of Stirling, Dementia Services Development Centre*].

** An asterisk indicates a Collaborative Partner, whose participation is on a voluntary basis. All other names indicate Associated Partners, who receive financial support from the European Commission*
