# Supplementary material for: Sharing knowledge to advance healthcare policies in Europe for people living with dementia and their carers: the ALCOVE project
Source: Arch Public Health. 2012 Aug 28;70(1):21. doi: 10.1186/0778-7367-70-21 (PMC3523028; doi:10.1186/0778-7367-70-21)
Supplement: Additional file 2 — Annex 2. ALCOVE QUESTIONNAIRES. [file 0778-7367-70-21-S2.doc]

**ALCOVE QUESTIONNAIRES**

**
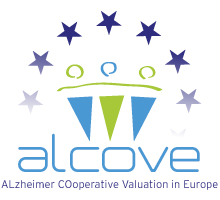
**

**DEMENTIA DIAGNOSIS**

***(respondent: MoH - Ministry of Health)***

**COUNTRY DATA**

In your country there are approximately how many of the following professionals?

| Neurologists |  |
| --- | --- |
| Geriatricians |  |
| Psychiatrists (in total) |  |
| Psychiatrists (specializing in old age) |  |
| General Practitioners/ Family Doctors |  |

Approximately what percentage of professionals work in different settings?

| Professional | Hospital | Ambulatory/ Community |
| --- | --- | --- |
| Neurologists |  |  |
| Geriatricians |  |  |
| Psychiatrists (in total) |  |  |
| Psychiatrists (specializing in old age) |  |  |

How many people are there in total of the following age in your country?

| Over 65 years old |  |
| --- | --- |
| Over 75 years old |  |
| Over 85 years old |  |

What is the average stage of the disease at the moment of diagnosis of dementia?

MMSE <10 (severe) MMSE 10-20 (moderate) MMSE 21-25 (mild)

Other (please specify):

Please specify the source of the data *(e.g. survey, health insurance, professional opinion, other...)*:

Approximately what percentage of diagnoses are missed? Please specify the source of the data

(e.g. survey, health insurance, professional opinion, other...):

**DEMENTIA DIAGNOSIS**

Does your country have national official guidelines for diagnosis (endorsed

by national agency or ministries)?

Please give a website link if applicable:­­­­­­­­

Which tools are most frequently used in day-to-day practice to detect early dementia?

MMSE Yes / No Clock drawing test Yes / No

MoCA Yes / No ACE R Yes / No

Other (please specify):

Do you have allied medical professionals to test memory? (Please tick ✓ all that apply)

| **Professional** | Yes, always | Yes, often | Yes, sometimes | No, never |
| --- | --- | --- | --- | --- |
| **Neuropsychologists** |  |  |  |  |
| **Clinical Psychologists** |  |  |  |  |
| **Occupational Therapists** |  |  |  |  |
| **Nurses** |  |  |  |  |
| **Psychiatrists** |  |  |  |  |
| **Geriatricians** |  |  |  |  |
| **Other (please specify):** |  |  |  |  |

How many of each kind of professional are there?

| Neuropsychologists |  |
| --- | --- |
| Clinical Psychologists |  |
| Occupational Therapists |  |
| Nurses |  |
| Other (please specify): |  |

Do you have screening services for: *(Please circle as appropriate)*

Dementia? ***Yes / No*** Mild Cognitive Impairment (MCI)? ***Yes / No***

*Where are the services located? (Please tick ✓* all that apply)

|  | Community based | In hospitals | In care homes | Not applicable |
| --- | --- | --- | --- | --- |
| Dementia |  |  |  |  |
| MCI |  |  |  |  |

What are the services for? *(Please tick ✓ all that apply)*

|  | General population screening | Screening of at-risk populations | Opportunistic screening | Not applicable |
| --- | --- | --- | --- | --- |
| Dementia |  |  |  |  |
| MCI |  |  |  |  |

**Theory vs Practice**

Regarding medical imaging in dementia, what is ***recommended*** for the following patient types?

Please complete the table using the following information: 1) routinely 2) for research purposes 3) for specific patients** 4) never

| Recommended (Theory) | No image | Computed Tomography (CT) scan | Magnetic Resonance Imaging (MRI) | Positron Emission Tomography (PET) and single-photon emission CT (SPECT) | Other*** |
| --- | --- | --- | --- | --- | --- |
| Young/working age (<65)* |  |  |  |  |  |
| Pre-dementia/ MCI |  |  |  |  |  |
| Early without obvious clinical signs |  |  |  |  |  |
| Early with obvious clinical signs |  |  |  |  |  |
| Late stage |  |  |  |  |  |

* If young/working age is different from <65, please specify:

** If for specific patients, please specify which ones:

*** If other images used, please specify:

Are the above practices recommended across all regions in your country?

Please circle: *Yes / No*

If No, please elaborate:

What evidence were the above practices based on? *(e.g. survey, legislation, professional opinion, other)*

Regarding medical imaging in dementia, what is ***actually implemented*** for the following patient types? (Please complete the table using the following information: 1) routinely 2) for research purposes 3) for specific patients 4) never

| Actually implemented (Practice) | No image | Computed Tomography (CT) scan | Magnetic Resonance Imaging (MRI) | Positron Emission Tomography (PET) and single-photon emission CT (SPECT) | Other |
| --- | --- | --- | --- | --- | --- |
| Young/working age (<65) |  |  |  |  |  |
| Pre-dementia/ MCI |  |  |  |  |  |
| Early without obvious clinical signs |  |  |  |  |  |
| Early with obvious clinical signs |  |  |  |  |  |
| Late stage |  |  |  |  |  |

What evidence were the above practices based on? *(e.g. survey, legislation, professional opinion, other)*

In your country are biomarkers measured in the cerebrospinal fluid (CSF): *(Please circle all that apply)*

Routinely? ***Yes / No*** For research purposes? ***Yes / No*** For specific patients? ***Yes / No***

Never? ***Yes / No*** If ‘For specific patients’ which ones?

Who is/are ***officially designated*** to carry out the following roles, and who carries them out ***in day-to-day practice/reality***? *(Please complete the table using the following information)*:

1) GP 2) Neurologist 3) Geriatrician 4) Psychiatrist 5) Other medical specialist 6) Occupational therapist 7) Psychologist 8) Nurse 9) No specific profession

| Role | | Clinician(s) officially responsible (Theory) | Clinician(s) actually performing role (Practice) |
| --- | --- | --- | --- |
| Assess intellectual functions including memory test | |  |  |
| Make a diagnosis | Overall |  |  |
|  | In simple cases |  |  |
|  | In complex cases |  |  |
| Disclose diagnosis to patients and care giver | |  |  |
| Determine an individual patient management strategy | |  |  |
| Implement early psychosocial interventions | |  |  |
| Initiate anti-dementia drugs | |  |  |
| Ensure the follow up of patients and carers | |  |  |
| Monitor anti-dementia drugs | |  |  |
| Monitor adverse drug reactions | |  |  |
| Discontinue treatment | |  |  |
| Monitor deterioration in cognitive function | |  |  |

Does your country offer any of the following psycho-social interventions **directly following diagnosis** for people with dementia and their families, and if so how often? *(Please ✓ all that apply)*

| Intervention | Yes, always | Yes, often | Yes, sometimes | No, never |
| --- | --- | --- | --- | --- |
| Information about dementia |  |  |  |  |
| Education about dementia |  |  |  |  |
| Social support |  |  |  |  |
| Financial advice |  |  |  |  |
| Cognitive Stimulation Therapy |  |  |  |  |
| Peer support groups such as Alzheimer’s cafes |  |  |  |  |
| Counselling services |  |  |  |  |
| Psychotherapy |  |  |  |  |
| Family support services |  |  |  |  |
| Reminiscence or life story work |  |  |  |  |
| Creative therapies such as art, music, dance |  |  |  |  |
| Other (please describe): |  |  |  |  |

**LEGAL FRAMEWORK**

Do you have legislation to protect vulnerable adults that includes people with dementia?

Do you have legislation for advance statements and advance directives?

If Yes, what does this legislation include? *(Please tick ✓all that apply)*

| Arrangements and wishes for the future | |  |
| --- | --- | --- |
| A power of attorney, which identifies a person who will make decisions about: | Health care |  |
|  | Finances |  |
| Aspects that require future plans | |  |
| Advance directives on end of life care | |  |
| Other (please specify): | |  |

Are the following professionals familiar with this legislation? *(Please tick ✓all that apply)*

| Professional | Yes, always | Yes, often | Yes, sometimes | No, never |
| --- | --- | --- | --- | --- |
| GPs |  |  |  |  |
| Specialists |  |  |  |  |
| Other (please specify): |  |  |  |  |

*Who encourages patients to design an advance directive and how often? (Please tick ✓*all that apply)

| Professional | Yes, always | Yes, often | Yes, sometimes | No, never |
| --- | --- | --- | --- | --- |
| GPs |  |  |  |  |
| Specialists |  |  |  |  |
| Other (please specify): |  |  |  |  |

**W**ho is responsible for the promotion of these legal provisions?

Any additional comments regarding legislation? *(Optional)*

**Health organisations**

Do you have specialist memory clinics (a clinical setup providing specific assessment consultations for memory or cognitive impairment)?

If Yes, how many are there in your country? If Yes, are the clinics:

Extensive/Widespread ***Yes / No***, Limited ***Yes / No****,* Only a few ***Yes /No***

Do you have other types of expert memory services (multi-disciplinary team, memory clinic, clinical research and training activities)? If Yes, how many are there of each type in your country?

If Yes, are the services:

Extensive/Widespread ***Yes / No***, Limited ***Yes / No****,* Only a few ***Yes /No***

What is the average waiting time from referral to see a specialist for a memory assessment?

Please specify the source of the data *(e.g. survey, professional opinion, other)*:

In your perception, is the memory assessment service accessible to all patients?

Approximately what percentage of your country is covered by:

| Memory centres? |  |
| --- | --- |
| Dementia specialists? |  |

Are there specific centres for younger/working age (<65) patients with suspected or established dementia?

Have there been communication campaigns organized to raise awareness about dementia?

If Yes, were the campaigns aimed at:

The general public? ***Yes / No*** Professionals? ***Yes / No***

Please give website link(s) if applicable:­­­­­­­­­

Does your country have specific policies to improve the quality of diagnosis?

If Yes, please provide details:

Are there examples of overarching/integrated dementia pathways from pre-diagnosis to end of life and beyond?

If Yes, please provide examples of good practice:

**Relationships between GPs and specialists**

*What kind of information or notification is the GP* ***supposed*** *to give to the specialist when referring a patient? (Please tick ✓* all that apply)

| Clinical history |  |
| --- | --- |
| Signs and symptoms |  |
| Blood screen |  |
| Urine screen |  |
| No specific information required |  |
| Other (please specify): |  |

What kind of information or notification is the specialist ***supposed*** to give to the GP when sending a patient back? *(Please tick ✓ all that apply)*

| Discharge summary |  |
| --- | --- |
| Cognitive assessment |  |
| Treatment proposal |  |
| Prognosis |  |
| No specific information required |  |
| Other (please specify): |  |

How often is this information ***actually*** shared between the GP and the specialist? *(Please tick ✓ as appropriate)*

| **Information flow** | **Always** | **Most of the time** | **Sometimes** | **Rarely** | **Never** |
| --- | --- | --- | --- | --- | --- |
| GP to specialist |  |  |  |  |  |
| Specialist to GP |  |  |  |  |  |

How long, on average, does the transmission of this information take?

| GP to specialist |  |
| --- | --- |
| Specialist to GP |  |

In your country is there specific training and/or accreditation to enable GPs to diagnose dementia?

In your country is there specific training and/or accreditation to enable GPs to recognise the symptoms of early dementia?

Approximately what percentage of GPs are trained to diagnose dementia?

Approximately what percentage of GPs are trained to recognise the symptoms of early dementia?

**Questionnaire: National programs dedicated to dementia**

***(respondent: MoH)***

Generally speaking, by **National Policy** we intend the stated principles which guide the actions of government. A public policy is a purposive and consistent course of action produced as a response to a perceived problem of a constituency, formulated by a specific political process, and adopted, implemented, and enforced by a public agency. A **National Program** usually, but not always, follows and translate into action a National Policy.

**SECTION 1. EXISTING DEMENTIA STRATEGY**

In your Country, is there a National Program or any written National policy on dementia health and social care services organization? (**If No, go to section 4)**

If *Yes*, please specify:

Title (in original language and English translation):

Date of issue: ____/____/_______ 1.2.3 Date of review (if any): ____/____/_______

Web site:

Is it a stand alone plan or included in more vast plan (i.e. mental health programme, etc)?

□ Stand alone plan □ Included in

Are there other Programs or policies on dementia care service organization at Sub-National-Local Government level (State, Region, Department)?

By sub-national government(s) we intend the lower(s) level(s) in administrative State organization having autonomy in decision-making and have some or the same kind of powers as national governments do (States, Regions, Department, other)

□ Yes, depending on the National Program □ Yes, independent Sub-National Programs □ No □ Other, specify

Has the National or Sub-National program been based on national/local data?

**SECTION 3. IMPLEMENTATION OF THE NP ON DEMENTIA**

Since its delivery, the National Program/Policy has been implemented?

□ Yes, completely □ Yes, partially, because □ No, because

How would you characterize the monitoring process?

|  | Yes | No | Not Appl. |
| --- | --- | --- | --- |
| A set of outcomes, standards and indicators have been defined | □ | □ | □ |
| Indicators are measured on a regular monitoring basis | □ | □ | □ |

**SECTION 4. STRUCTURES AND SYSTEMS DEDICATED TO BPSD**

How would you characterize the structures and systems dedicated to BPSD?

|  | Yes | No | Estimated general population coverage % |
| --- | --- | --- | --- |
| Existing Mobil team (mental health, geriatrics, multi disciplinary, etc.) | □ | □ | …. |
| Integrated care and case management | □ | □ | …. |
| Day hospitalization dedicated to BPSD | □ | □ | …. |
| Respite unit | □ | □ | …. |
| Dedicated unit in Nursing homes | □ | □ | …. |
| Dedicated units in hospitals …………  ………………………………………..…Fully dedicated to BPSD (AD)  ………………………………………..…Psychiatry unit  ………………………………………..…Neurology unit  ………………………………………..…Geriatrics unit | □  □  □  □ | □  □  □  □ | …  …  …  … |

**SECTION 2. COMPONENTS OF THE NP ON DEMENTIA**

The National Program/Policy explicitly provides general statements and/or recommendation for:

At the end of this questionnaire, we would like to know your own opinion on the NP. Notice that what you will write in this section will be kept anonymous and used in aggregate form only for qualitative analysis purpose.

**Questionnaire: Available data & databases on dementia and on health/social services dedicated to dementia**

***(respondent: MoH)***

***OVERVIEW OF ALL AVAILABLE DATA SOURCES for the purpose of the dementia study***

| Description of the data source* *Please, add rows if needed.* | Name of the data source (in original language and English translation) | Contact person | **Institution** | **Mail / Tel / Fax** |
| --- | --- | --- | --- | --- |
|  |  |  |  |  |
|  |  |  |  |  |

*A source of data may be a centralized computerized medical file, personal computerized file, drugs file, reimbursement databases (claim data), hospital administrative database, nursing home-residential care respite unit, death national registry, cohorts, other registries, surveys or other data sources specific for each Country. **Please, feel free to add data sources type according to your Country system**.

**Questionnaire: databases characteristics**

***(respondent: referent of the database)***

**DATA SOURCE**

*Please indicate the source of data you are describing, choosing among the proposed categories. If not included, add an “other” category.*

- Computerized medical file (centralized at national or sub-national level)
- Personal computerized file
- Drugs file
- Reimbursement database (claim data)
- Hospital administrative database
- Nursing home - residential care respite unit information
- Death national registry
- Cohorts, registries
- Surveys
- Other, specify ______________________________
- **Name**

*Name of the data base in original language* **AND** *English translation*

- **Original purpose**

*Indicate the original purpose of the data (i.e. demographic survey on general population, surveillance system on ageing, etc)*

- **Use for dementia**

*Indicate what use is done or could be done for the purpose of epidemiology, health and social services related to dementia*

- **Please, describe benefits, limitations and suggestion on the use of this data source for the purpose of dementia study**

**BENEFITS** Indicate the advantages of this data source for dementia study purpose:

**LIMITATIONS** Indicate the limitations of this data source for dementia study purpose:

**SUGGESTIONS** Indicate your suggestions to improve this data source for dementia study purpose:

**DATA TYPE**

*Describe the type and nature of data used (i.e. reimbursement for medications, diagnosis in medical records, etc)*

- **Quantity**

*Indicate numerical data, define when possible numerator and denominator*

Numerator definition:

Denominator definition:

**Years** *Indicate the year or range of years of reference for previous data*

- **Used for**

*Please choose type of data among the proposed categories*

- Diagnosis AD & other dementia
- Treatment (antipsychotics)
- Social data
- Other clinical data
- Other, specify

***Are there other data used from the same data source? If yes, please continue.***

**Questionnaire: Available data about prescriptions of psychotropic medications in dementia**

***(respondent: MoH)***

- Are the medications prescribed paid by the Health System?

□ Yes □ No □ Partly

- Is it possible to collect specific information about prescriptions of cholinesterase inhibitors and/or memantine?

□ Yes □ No

- If the answer to question (2) is yes, are pharmacoepidemiological indicators available?

□ Yes □ No

- If the answer to question (3) is yes, please specify both qualitative and quantitative data (i.e. DDD, prevalence of use, etc.)
- Are these data:
- Census based (population based) □Yes □No
- Sample data (based on defined sample methodology) □Yes □No

What percentage of the total population is covered? If the coverage is incomplete, are the data extrapolated to reach 100% coverage? □Yes □No if yes please specify:

- Are there data available on the prescriptions of psychotropic medications in these specific settings?
- Outpatient clinic □Yes □No if yes please specify:
- Hospitals □Yes □No if yes please specify:
- Nursing home □Yes □No if yes please specify:
- Population □Yes □No if yes please specify:
- Is it possible to ascertain if a prescription for a psychotropic medication is made for a person affected by dementia? □Yes □No

If yes please specify for the following categories:

- Antidepressants (ATC N06A):
- Benzodiazepines (ATC N05B):
- Antipsychotics (ATC N05A):
- **Questionnaire: Clinical guidelines on behavioral and psychological symptoms of dementia (BPSD)**

***(respondent: MoH)***

Clinical guidelines, clinical care guidelines or clinical practice guidelines are recommendations on the appropriate treatment and care of people with specific disease or condition. They are based on the best available evidence. While clinical guidelines help health professionals in their work, they do not replace their knowledge and skills (NICE).

- Does your Member State have a generally accepted and generally used (national) clinical guideline(s) on BPSD or a clinical guideline that addresses BPSD in conjunction with another related problem, for example memory disorder? YES/NO
- How is it (are they) entitled? Please provide an English translation of the title and a few sentences on the content of the guideline. In addition, please describe who are responsible/authors of the guideline, the year it was issued and some general characterizations of methodology of the guideline (for example an expert opinion, peer-reviewed, evidence based, panel of experts, Delphi-method etc.) .
- Does the clinical guideline include psychosocial (otherwise also known as non-pharmacological) interventions on BPSD? YES/N
- If YES, please describe the general message of the guideline regarding the psychosocial interventions on BPSD in few sentences.
- Does the clinical guideline include pharmacological interventions on BPSD? YES/NO
- If YES, please describe the general message of the guideline regarding the pharmacological interventions on BPSD in few sentences.
- In addition, please describe in detail how the guideline instructs the use of antipsychotic drugs in treating BPSD.
- Does the clinical guideline include recommendations for social support for patients with BPSD or their care givers? YES/NO
- If YES, please describe the general message of the guideline regarding the social support for patients with BPSD or their care givers in few sentences.
- Please give your opinion on the guideline(s). Do you think it is (they are) generally accepted and considered reliable? Is it up to date or should it be updated?
- The future of the BPSD related guidelines in your Member State: Are there new recommendation(s) in the making or are the current one(s) being updated or modified? Are there other news regarding BPSD guidelines?

**Questionnaire: Assessment of the support systems for BPSD**

***(respondent: referent of each structure)***

**QUESTIONNAIRE: Assessment of the support programs for caregivers to prevent BPSD**

***(respondent: referent of each support programs)***
